# Is the Exposome Involved in Brain Disorders through the Serotoninergic System?

**DOI:** 10.3390/biomedicines9101351

**Published:** 2021-09-29

**Authors:** Denis Sarrouilhe, Norah Defamie, Marc Mesnil

**Affiliations:** 1 Laboratoire de Physiologie Humaine, Faculté de Médecine et Pharmacie, 6 Rue de la Milétrie, Bât D1, TSA 51115, CEDEX 09, 86073 Poitiers, France; 2 Laboratoire STIM, ERL7003 CNRS-Université de Poitiers, 1 Rue G. Bonnet–TSA 51106, CEDEX 09, 86073 Poitiers, France; norah.defamie@univ-poitiers.fr (N.D.); marc.mesnil@univ-poitiers.fr (M.M.)

**Keywords:** brain, glioblastoma, neurobehavioral disorders, neurodegenerative disorders, neurodevelopmental disorders, pesticides, pollutants, serotonin

## Abstract

Serotonin (5-hydroxytryptamine, 5-HT) is a biogenic monoamine acting as a neurotransmitter in the central nervous system (CNS), local mediator in the gut, and vasoactive agent in the blood. It has been linked to a variety of CNS functions and is implicated in many CNS and psychiatric disorders. The high comorbidity between some neuropathies can be partially understood by the fact that these diseases share a common etiology involving the serotoninergic system. In addition to its well-known functions, serotonin has been shown to be a mitogenic factor for a wide range of normal and tumor cells, including glioma cells, in vitro. The developing CNS of fetus and newborn is particularly susceptible to the deleterious effects of neurotoxic substances in our environment, and perinatal exposure could result in the later development of diseases, a hypothesis known as the developmental origin of health and disease. Some of these substances affect the serotoninergic system and could therefore be the source of a silent pandemic of neurodevelopmental toxicity. This review presents the available data that are contributing to the appreciation of the effects of the exposome on the serotoninergic system and their potential link with brain pathologies (neurodevelopmental, neurodegenerative, neurobehavioral disorders, and glioblastoma).

## 1. Introduction

Serotonin (5-hydroxytryptamine, 5-HT) is a biogenic monoamine that acts as a neurotransmitter in the central nervous system (CNS), a hormone in the gut, a mitogen factor, and that can regulate vascular tone [1]. A two-step synthetic pathway from the essential amino acid tryptophan involving the rate-limiting enzyme tryptophan hydroxylase (TPH1, mostly expressed in the periphery, and TPH2, the neuron-specific isoform), and then 5-hydroxytryptophan (5-HTP) decarboxylase, produces serotonin. In the CNS, serotonin is concentrated in the synaptic vesicles of neurons by the vesicular monoamine transporter (VMAT), and removed from the synaptic cleft by the selective serotonin transporter (SERT, SLC6A4), and further recycled either back into presynaptic vesicles or metabolized to 5-hydroxyindole-3-acetic acid (5-HIAA) mainly by monoamine oxidase-A (MAO-A) within the neuronal cytosol [2]. In the CNS of vertebrates, a majority of the cell bodies of serotoninergic neurons is in the raphe nuclei of the brain stem. The neurons of the raphe nuclei give rise to broad projections to the forebrain (rostral group) and to the hindbrain (caudal group), allowing serotonin to influence many brain functions [3]. In humans, 13 receptor subtypes are recognized, spreading over seven receptor families, with different gene splice variants for some of them [4]. Except for the 5-HT3, which is a ligand-gated ion channel, all the serotonin receptors belong to the family of G-protein coupled receptors that allows serotonin to modulate the activity of different effector systems, such as adenylyl cyclase and phospholipase C [5,6]. In addition, many subtypes of serotonin receptors can modulate the activity of ERK1/2 and Akt [7,8]. Serotonin, which is now considered a neurohormone, has been linked to diverse CNS functions and is implicated in many CNS disorders, including neurodevelopmental, neurodegenerative, neurobehavioral diseases, and cancer (glioma) [9].

In the CNS, serotonin is a morphogenic agent and a neurotrophic factor directing brain development during embryogenesis [10]. However, in mice and humans, before the formation of the dorsal raphe, the placenta is the source of serotonin for the early forebrain development [11]. In humans, the two first trimesters of development include cortical neurogenesis, migration, and initial axon targeting [12]. A recent study reveals that ex vivo activation of the 5-HT2A receptor in the fetal human neocortex promotes basal progenitor proliferation, cells that are linked to mammalian neocortex evolutionary expansion [13]. In mice, forebrain disruption of serotonin signaling affects axon guidance leading to abnormal thalamocortical axon trajectories [14]. Moreover, in the first postnatal week of the rodent, the serotoninergic system has a transient influence on the development of the barrel fields in layer IV of the somatosensory cortex [15].

During the last decades, the serotoninergic system emerged as a target of an increasing number of environmental pollutants. Among them are pesticides, bisphenol A (BPA), phthalates, polychlorinated biphenyls (PCBs), polycyclic aromatic hydrocarbons (PAHs), fine particulate matter with a diameter less than 2.5 µm (PM_2.5_), and toxic heavy metals. During prenatal development, before the maturation of the blood-brain barrier (BBB), pollutants can exert their toxic effects on neural tissue. The developing nervous system of a fetus and newborn is susceptible to the deleterious effects of neurotoxic substances in our environment, and perinatal exposure could result in the later development of diseases [16]. From childhood up to adult life, because of their lipophilic properties, some of these pollutants are able to cross the BBB and reach the CNS [17]. This review presents the data that are contributing to establish a link between the effects of the exposome on the serotoninergic system and the increasing incidence of brain pathologies.

## 2. Effects of Environmental Chemical Pollutants on the Serotoninergic System

**Figure 1:**
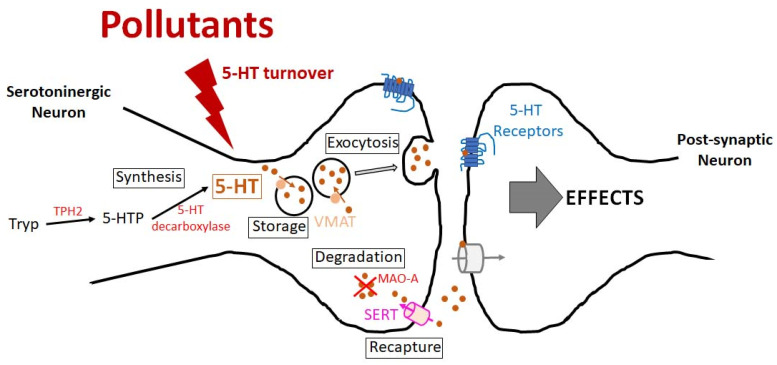
Effects of environmental chemical pollutants on the serotoninergic system. This figure summarizes the potential action of chemical pollutants on serotonin turnover. Other reported actions are on the expression of serotonin transporter genes and on the expression of serotonin receptor subtypes. See text for details. 5-HT: 5-hydroxytryptamine, serotonin; 5-HTP: 5-hydroxytryptophan; MAO-A: monoamine oxidase-A; SERT: serotonin transporter; Tryp: tryptophan; TPH2: tryptophan hydroxylase; VMAT: vesicular monoamine transporter.

BPA (2,2-bis(4-hydroxy-phenyl)propane) is a synthetic estrogen that was widely employed in a variety of consumer products made of polycarbonate plastic and epoxy resins. Exposure to BPA in humans is widespread and almost continuous, and this pollutant has been shown to be transferred to the CNS via the BBB [18]. The effects of BPA can be mediated by classical nuclear estrogen receptors (ERs), non-nuclear ERs, and also by the seven-transmembrane GPR30 ER and the estrogen-related receptor γ [17]. Fetal, prenatal, and lactational BPA exposures were suggested to perturb the serotoninergic system in adult rodents [19]. Thus, studies conducted in rats and mice suggest that BPA increases the turnover of serotonin in the brain (Figure 1) [20,21,22]. In the hippocampus of the young female mice, this change in the turnover is mediated by an increased gene expression of enzymes of the serotonin metabolism (Tph2 and Maoa) and its carrier (SLC6A4) [23]. On the other hand, mouse prenatal and lactational BPA exposure does not alter serotoninergic neurons’ immunoreactivity and morphology in the dorsal raphe [24]. Long-term exposure to BPA also disrupts serotonin levels in the forebrain of adult male rats through changes in its metabolism [25]. Two substitutes of BPA, bisphenol F and bisphenol S, also affect the serotoninergic system in the prefrontal cortex of juvenile female rats, highlighting the importance of preventive vigilance in the industrial use of these compounds [26].

Pesticides are a heterogeneous group of chemical substances used to eliminate pests and to protect crops with many different mechanisms of action. Based on the types of targets, pesticides include herbicides, which are the most common, insecticides, antiparasitics, and fungicides. In developing rat brains, the serotoninergic system is vulnerable to disruption by organophosphate insecticides altering the architectural assembly of the brain and later behavior. However, three widely used organophosphate pesticides, chlorpyrifos, diazinon, and parathion, showed a distinctly different spectrum of actions on TPH induction, expression of serotonin transporter genes, and expression of serotonin receptor subtypes [27,28], pointing to mechanistic differences between their effect on neural development [29]. The organochlorine insecticide dieldrin strongly induces TPH and has a similar effect to that of diazinon on the pattern of expression of serotonin receptors [28]. The organochlorine insecticide lindane (γ-hexachlorocyclohexane) enhances serotonin levels in brain mice. Several mechanisms have been proposed to explain this observation, either a direct action on serotoninergic neurons or an indirect action, by an effect on GABAergic inhibitory neurons. Indeed, the treatment of mice with lindane results in a decrease in the levels of GABA in the nervous system by inhibition of glutamic acid decarboxylase (GAD). Thus, the action of lindane would raise a tonic inhibitory action exerted on serotoninergic neurons [30]. Another organochlorine insecticide, 1,1,1-trichloro-bis(p-chlorophenyl)ethane (p,p′-DDT) p,p′-DDT, induces a marked increase in 5-HIAA, but not in serotonin, in the brain stem, hypothalamus, and striatum of rats [31]. The organochlorinated chlordecone (kepone) follows several toxicological pathways, including in vivo neurotoxicity and perturbation of the serotoninergic system [32]. Increases in both serotonin and 5-HIAA content, and only in serotonin content, were detected in the hypothalamus and the preoptic area of chlordecone-treated female rats, respectively [33]. The chronic administration of fipronil, a broad-spectrum insecticide that belongs to the phenylpyrazole chemical family, induces massive and inhomogeneous changes in the serotoninergic systems in the rat brain [34]. Type II pyrethroids (with an α-cyano group) accelerated the turnover of serotonin in the midbrain and striatum areas of rats and so can affect serotonin neurotransmission levels [35].

Polychlorinated biphenyls (PCBs) are industrial chemical mixtures used in a wide variety of commercial applications. Increased serotonin levels were observed after exposure of rats to PCB153 [36].

Phthalates are dialkyl or alkyl aryl ester derivatives of phthalic acid (1,2-benzenedicarboxylic acid) that are used in a variety of products to make them flexible and soluble, including cosmetic products and lotions, aerosol delivery agents, plasticizers and adhesives, flooring and medical tubing [37]. Phthalates have estrogenic activity and can interfere with nuclear receptors, membrane receptors, intracellular signaling pathways, and modulate gene expression [38]. Upregulation of some serotonin receptor subtypes was observed after rat exposure to the phthalate di-cyclohexylphthalate (DCHP) [39]. Benzyl butyl phthalate (BBP) was also reported to modulate the serotoninergic system in the brain of Fundulus heteroclitus (mummichog) [40]. Interestingly, a recent study that better mimics the real-life situation demonstrated that administration of a mixture of phthalates, pesticides, and BPA to mice throughout gestation modified serotonin receptor subtypes 1A and 2A expression in the limbic system of adult offspring animals [41]. These two serotonin receptor subtypes were known to be involved in emotionality and stress coping strategies [42].

Ambient and household air pollution have an increasing impact on public health in industrial countries. Fine and ultrafine particulate matter (PM_2.5_, less than 2.5 and PM_0.1_, less than 0.1 µm in diameter, respectively) can translocate to the brain either through the BBB [43] or via the olfactory epithelium [44] or via sensory afferents in the gastrointestinal tract [45]. Lower levels of the serotonin metabolite 5-HIAA were found in human adults exposed to urban air pollutants [46]. Diesel exhaust nano-sized particles are among the most abundant air pollutants in urban environments. Prenatal exposure to diesel exhaust particles decreases the serotonin and 5-HIAA levels in the nucleus accumbens, amygdala, and hypothalamus of male mice [47]. Ozone (O_3_) exposure in rats changes the pattern of serotonin receptors expression in the hippocampus [48] and reduces serotonin levels in the frontal cortex and hippocampus [49].

## 3. Neurodevelopmental Disorders

The etiology of autism spectrum disorders (ASD) is believed to involve genetic, epigenetic, and environmental components [50]. In patients with ASD, an increased whole-blood serotonin level and dysfunction of the brain serotoninergic system have been described even if a clear link cannot be established between the two phenomena [51]. A hyperserotonemia is also found among some parents, brothers, and sisters of ASD children, suggesting the involvement of genetic susceptibility factors related to the serotoninergic system in ASD [52,53]. At the brain level, studies using medical imaging showed a difference in serotonin synthesis capacities, focal and asymmetrical serotonin synthesis abnormalities, and decreases in serotonin transport and binding to its receptors in ASD children compared to control children [54,55]. It is interesting to note that serotonin levels have an influence on the development and size of the barrel fields and that alterations in the organization of cortical columns have been detected in ASD [15,56]. Several studies point to the importance of serotonin for social function, cognitive flexibility, stereotypic behavior and sensory development, modulation of the processing of facial expressions of emotion, sleep-wake rhythm, and locomotion, phenomena that significantly differ in ASD patients compared with healthy control individuals [57,58,59]. Moreover, the CHARGE study showed that in boys, prenatal exposure to selective serotonin reuptake inhibitors (SSRI) such as fluoxetine, especially during the first trimesters, may be associated with an increased risk of ASD [60]. There is evidence suggesting that a number of environmental pollutants (BPA, pesticides, traffic-related air pollution, phthalates, …) contribute to ASD pathogenesis [61]. The CHARGE study reports an increased risk of ASD diagnosis among children whose mothers lived during pregnancy near fields where pesticides, particularly organophosphates, were applied [62]. A positive association was recently corroborated in the SEED study between air pollution exposure during the late prenatal and early postnatal periods and ASD [63]. Moreover, there is increasing concern that BPA exposure may influence human brain development and contributes to the increasing prevalence of ASD. For the first time, a study of 46 children with ASD and 52 controls found a direct association between children with ASD and BPA exposure and demonstrated that children with ASD do not metabolize BPA correctly. The metabolomic analyses showed a correlation between ASD and essential amino acid metabolism pathways such as tryptophan, the serotonin precursor [64]. The aryl hydrocarbon receptor (AhR) could represent an additional level of interaction between BPA and the serotoninergic system. Indeed, BPA and some tryptophan catabolites (TRYCATs) are AhR ligands, and some of them are produced by the commensal microbiome whose involvement has been proposed in the development of ASD [65,66]. Altogether, these results suggest that the link between BPA and ASD could be a defect of in utero or perinatal serotoninergic system development or function [67].

Attention deficit hyperactivity disorders (ADHD) etiology is multifaceted, with many risk factors, including prenatal and perinatal expositions to environmental toxins, even at exposure levels considered safe for adults. Organophosphate pesticides, PCBs, lead, BPA, phthalates, and air pollution exposition have been associated with an increased risk of ADHD [68,69,70,71,72,73]. These agents may have a neurotoxic effect on the neural systems involved in ADHD [74], in particular the serotoninergic system. In rat and mouse models, fetal and prenatal BPA exposure was suggested to perturb the serotoninergic system [20,21,22], which is suspected to be involved in ADHD etiology [75]. A complex gene-environmental toxins interplay could amplify ADHD risk early on in life through epigenetic mechanisms [76]. Genetic studies identified candidate ADHD risk genes [77] such as those associated with the serotoninergic system (SLC6A4, coding for SERT; HTR1B, HTR2A, coding for the 1B and 2A serotonin receptors; DDC, coding for dopamine decarboxylase; TPH2). Serotonin deficits have been proposed to be involved in the etiology of the hyperactive and impulsive component of ADHD. Interestingly, oral administration of the precursor of serotonin, tryptophan, allowed significant improvement of ADHD symptoms [78]. A recent case-control study made on 216 students and strictly matching age, sex, height, weight and class, associated ADHD with low blood levels of serotonin. Therefore, the lack of impulse control and the aggressiveness found in ADHD may be partially related to lower blood levels of serotonin [79].

Pollutant exposure during pregnancy or after birth may be at the origin of epilepsy [17]. The link underlying this association is not understood but might be mediated by serotonin levels since its increase appears to be protective against seizures and sudden unexpected death in epilepsy (SUDEP) [80]. Similarly, animal models suggest that serotonin depletion is a risk factor for epilepsy [81]. This situation is in line with studies showing that seizures and epilepsy may reduce serotonin levels and increase the risk of both seizures and SUDEP [82,83]. Therefore, any environmental exposure leading to a decrease in serotonin is susceptible to lead to an increased risk of epilepsy. This link between serotonin levels and epilepsy occurrence is illustrated by the fact that mediators of serotonin function are also involved in epilepsy. For instance, patients with temporal lobe epilepsy (TLE) exhibit decreased binding to 5-HT1A receptors within several parts of the brain [80]. Studies in epilepsy patients have also shown that seizure-induced decrease in expression of SERT contributes to reduced serotonin reuptake [84,85]. SERT binding is also reduced within the neocortex of post-mortem samples from TLE patients [86]. Moreover, seizures may influence levels of serotonin metabolites such as 5-HIAA, which is decreased in the cerebrospinal fluid (CSF) of adults with progressive myoclonic epilepsy [87,88]. Pediatric epilepsy patients also exhibit decreased concentrations of tryptophan within blood serum and CSF [89,90]. Pollution leads to neuroinflammation, which may play a role in epilepsy. In this situation, leukocytes and inflammatory mediators seem to contribute to a reduction in seizure threshold [91]. Even if their significance is unknown, immune cells from patients with TLE with hippocampal sclerosis exhibit high expression of 5-HT1A, 5-HT1B, 5-HT2A, and 5-HT4 receptors [91]. Platelets are also probably involved in this process by secreting proinflammatory mediators during neuroinflammation and traumatic brain injury (TBI). These factors increase the permeability of the BBB, which may create a predisposition to epileptic seizures, as observed in a mouse model. In this model, it is interesting to note that if platelets contribute to increased BBB permeability and are present in the CNS parenchyma during epileptic seizures, they also secrete serotonin [92]. Apparently, the presence of platelets in the CNS parenchyma is sufficient to induce severe seizures, as shown by intracranial injections of platelets that mimic TBI-associated bleeding [92]. Therefore, the role of serotonin might be different in the neuroinflammation context by favoring the risk of epilepsy.

## 4. Neurodegenerative Disorders

Brain neurodegenerative disorders (Alzheimer’s disease (AD), Parkinson’s disease (PD), amyotrophic lateral sclerosis, Friedreich’s ataxia, Huntington’s disease, …) constitute a broad corpus of diseases [93]. All of them share neuron degeneration as a common characteristic leading to overlapping clinical features such as cognitive impairment, movement disorders (called ataxias), sleep disorders, and neuronal pathway alterations (protein quality control, autophagy–lysosome pathway, mitochondria homeostasis, protein seeding, propagation of stress granules, and synaptic toxicity and network dysfunction) [94]. However, the prevalence of each of them varies greatly and affects people in an age-related manner. Rare neurodegenerative disorders (for example, amyotrophic lateral sclerosis, Friedreich’s ataxia, Huntington’s disease) mostly affect young people, whereas PD and AD affect older people and are more prevalent in countries with high life expectancy. Such epidemiological differences seem to be the consequence of genetic causes. Indeed, genetic involvement is clearly established in cases of rare neurodegenerative disorders. If genetic involvement cannot be excluded for AD and PD, especially for younger cases, these pathologies are clearly associated with age in the general population and thus potentially with environmental exposure [95]. Therefore, due to their high prevalence in the human population and their increased risk with age [96], we will focus on the two most prevalent neurodegenerative diseases, PD and AD.

PD is a progressive neurodegenerative disorder characterized by selective degeneration of dopaminergic neurons in the substantia nigra, leading to a reduced level of dopamine in the cortex. It remains unclear whether dopaminergic neuronal death results from events triggered during development into adulthood or represents a cumulative effect throughout life. Although advanced age is the only unequivocally accepted risk factor, it has been postulated that exposure to environmental neurotoxins combined with aging could increase the risk of developing PD. Among those neurotoxins are pesticides (rotenone, paraquat, maneb, ziram). In rats, motor and depressive behaviors associated with serotonin and norepinephrine alterations induced by the administration of rotenone were observed [97]. As a major comorbidity of PD, depression is associated with the loss of serotoninergic neurons in neuronal cultures of the midbrain. The depolymerization of microtubules induced by rotenone or colchicine caused an accumulation of vesicles in the soma and killed the serotoninergic neurons by a mechanism dependent on the metabolism of serotonin in the cytosol [98]. Finally, it has been recently shown that the first signs of PD can appear in the gastrointestinal (GI) tract and in the olfactory system, preceding the onset of motor disturbances by several years. A study showed the presence of specific deficits in olfactory function associated with a concomitant decrease in tyrosine hydroxylase-positive neurons and an increase in the turnover of serotonin in the olfactory bulb. These results suggest that exposure to rotenone induces GI and olfactory dysfunction involving immunological and neurotransmitter alterations, similar to the early signs of PD. This provides further evidence for the involvement of the gut-brain axis in PD [99]. Paraquat, a widely used herbicide in the world, leads to the apoptosis of dopaminergic cells [100]. In addition, paraquat, in combination with other pesticides (maneb and ziram), increased synergistically three times the risk of developing PD [101]. One study hypothesized that exposure to paraquat and maneb during critical periods of development could permanently alter the nigrostriatal dopamine system. These results indicate that exposure to the mixture of the two pesticides during the postnatal period may produce permanent and progressive damage to the nigrostriatal dopamine system [102]. In addition, it has been shown that paraquat triggers processes characteristic of the early stages of degeneration of dopaminergic neurons and activates compensatory mechanisms involving dopaminergic, noradrenergic, serotoninergic, and GABAergic transmissions [103]. Biochemical analysis showed that paraquat and maneb reduce the tissue content of striatal dopamine alongside changes in the activity of subthalamic nucleus neurons without changing the content of norepinephrine and serotonin in the cortex [104]. Like other environmental neurotoxicants, ziram can enter the CNS from the nasal mucosa via the olfactory nerves. This is consistent with the evidence that exposure to dimethyldithiocarbamate (NaDMDC) increases the risk of PD and points to the possibility that the olfactory system may be a major pathway for entry of NaDMDC into the CNS [105].

AD has been reported to be the consequence of various risk factors such as genetic predisposition, obesity, smoking, diabetes, and exposure during life to environmental agents [106,107]. If genetic predisposition is considered to account for most cases (70%) [106,107], the part due to pollutant exposure is probably underestimated. Indeed, toxic metals (aluminum, copper, …) [108,109,110,111,112,113], pesticides (organochlorine and organophosphate insecticides: β-hexachlorocyclohexane, dieldrin, etc.) [114,115,116,117,118,119], industrial pollutants (flame retardants, BPA, phthalates, …) [120,121,122], airborne particles (PM_2.5_ and PM_10_) [123,124,125,126,127,128] and O_3_ [127,128] have been hypothesized to induce or aggravate AD. Despite their chemical and physical variety, these pollutants seem to act through a common process, neuroinflammation, due to microglia activation, which is known to play an essential role in neurodegenerative diseases such as AD and PD [124,125,129,130,131]. Activated microglia are known to release proinflammatory factors, such as TNFα and IL-1β [132,133], which are found to be increased in the CSF of patients with AD [134,135,136]. Neuritic plaques composed of Aβ and neurofibrillary tangles are, indeed, surrounded by astrocytes and microglia with reactive characteristics [137]. Interestingly, proinflammatory factors have been observed in biological samples (blood, urine, and necropsy tissue) of children and adults from polluted areas [131,138,139,140] and were related to amyloid processing (tau hyperphosphorylation, Aβ immunoreactivity, and plaques) and inflammation response in the human brain [138,139,140,141]. Similar observations linking brain damage (white matter lesions, damaged BBB, degenerating neurons) and neuroinflammation were made in dogs from highly polluted urban areas compared to dogs living in rural areas [142,143,144]. All these observations have been confirmed by experimental data obtained from rodent models. Indeed, such data demonstrate that PM (PM_0.1_, PM_2.5_) exposures elicit increased brain inflammation, measured by IL-1β and TNFα [145,146,147,148]. Such a phenomenon is accompanied, for longer exposures (30–39 weeks), by brain damage (loss of dendritic spine density in the CA1 region of the hippocampus) and buildup of Aβ plaques, which correlates with impaired cognitive outcomes [149,150]. Other kinds of exposures, as diesel exhaust particles or nickel nanoparticles, also lead to increased inflammatory cytokines (TNFα, IL-1β) and increased levels of Aβ42 in multiple brain regions of rats [151,152] and mice [153], suggesting that the effect on Aβ buildup in the brain may be, in part, due to the concentration of particulates exposed, rather than its chemical constituents. Globally, all these findings demonstrate the association between chronic exposure to PM and inflammation and the development of AD-like neuropathology. Interestingly, the use of transgenic mice also confirmed that PM exposure effects on AD pathogenesis can be increased with susceptible genotypes, as seen in epidemiological studies [154,155]. A possible link between neuroinflammation and AD could be mediated by the attachment of complement proteins, such as complement C3, which helps microglia in the clearing of the plaques and is up-regulated in AD, contributing to the synapse loss that leads to cognitive decline [156,157]. It was demonstrated that knocking out the gene of this molecule in mice models of AD improved the animals’ performance in both learning and memory tests, despite them having more plaques in their brains and fewer activated microglia [158]. Increased proinflammatory cytokines in AD, such as IL-1β and TNFα, impact the serotoninergic system by increasing the uptake rate of serotonin [159] through SERT [160]. Therefore, such an effect could lead to decreased serotonin levels, which might be related to depression that is currently observed in AD patients. Such a hypothesis is supported by studies reporting that accumulation of Aβ oligomers and toxins present in AD patients leads to depressive episodes in mice through microglial activation, alterations in the TNFα signaling pathway, and reduced presence of serotonin in the brain [161]. Interestingly, it has been shown that treatments with SSRIs reduce the number of cytokines in the circulation [162,163]. Moreover, the following increased levels of serotonin resulted in lower Aβ production, supporting the idea that serotonin-induced pathways influence Aβ deposits in a negative way [164]. Such an effect is probably linked with the fact that serotonin can prevent the activation of microglial cells that are induced by Aβ [161]. Therefore, the modulation of the serotoninergic system may represent a therapeutic target for AD treatment, as suggested by recent clinical data [165].

To summarize, it does not seem that pollutant exposure induces AD by directly impairing the serotoninergic system. According to our current knowledge, pollutants first activate neuroinflammation in the brain, which, in turn, leads to brain damage. Among such damage, the serotoninergic dorsal raphe nucleus can be one of the first brain locations to be affected by tau protein abnormalities [166] even if the degeneration of the serotoninergic system can be observed in other brain regions (cortical, striatal, thalamic, and limbic regions) of patients with cognitive impairments compared to cognitively normal controls [167].

## 5. Neurobehavioral Disorders

Behaviors result from a complex interaction of genetic, environmental, and psychosocial influences and different kinds of stressors. Neurobehavioral disorders, among which major depression is the most common mood disorder, may be seen in association with brain disease (e.g., stroke, multiple sclerosis (MS), dementia, and neuro-oncological conditions), brain impairment (e.g., metabolic and toxic encephalopathies), and/or injury [168]. The interactions of the population with different factors, including environmental pollutants, can potentiate these behavioral disorders.

Mood and emotional disturbances are common complications observed in post-stroke patients [169] and may manifest when the lesions damage the serotoninergic neuronal system. Accordingly, SSRIs are the first-line medication choice to treat depressive symptoms in stroke patients and generally improve mood symptoms [170]. Emerging data suggest a role of serotonin in the recovery of neurological dysfunction in stroke patients, but the efficacy of SSRIs to improve emotional disturbances and/or neurological dysfunction may depend on SERT gene polymorphisms [171].

MS is a progressive neurological disorder in which environmental and genetic etiologies were suspected. In this disease, the immune system attacks and destructs the myelin protective sheath that covers nerve fibers resulting in CNS dysfunction. Both synthesis and metabolism of serotonin are disrupted in the brain of patients with MS. The level of tryptophan is reduced in the plasma and the CSF of patients, changes that might lead to impaired synthesis of brain serotonin [172,173]. On the other hand, a low level of 5-HIAA was found in CSF of patients with MS [174]. In a proof-of-concept study, the SSRI fluoxetine has a neuroprotective effect by reducing the formation of new lesions in non-depressed MS patients [175]. Nevertheless, depression is a common comorbidity observed in MS, and dysregulation of the serotoninergic system is observed in both diseases. Thus, reregulation of the serotoninergic system with SSRIs was also effective in MDD treatment in MS patients [176,177].

Disruption in the serotoninergic system has been established in AD and related dementia (see supra, AD paragraph). However, new results of a brain scan study suggest that serotonin loss is a key player in cognitive decline rather than a byproduct of AD and other dementias. Compared to controls, the brain of patients with mild cognitive impairment had up to 38% less SERT, a selective marker of the integrity of the serotoninergic system. This observation could also explain the limited success of the treatment of AD with SSRIs in some studies [167]. AD and depression have a complex relationship. Early onset depression is an etiological risk factor for AD, and late-onset depression may be a catalyst of cognitive decline. Thus, taking into account that SSRIs have an impact on plaque formation rather than on plaque clearance, it has been suggested to use these antidepressants early in order to alleviate the risk of developing AD and to treat depression as a risk factor [178].

In neuro-oncology, serotonin, which is part of the tumor microenvironment, can contribute to gliomagenesis, and the serotoninergic system may represent a potential novel therapeutic target for the most common primary malignant brain tumor glioblastoma (GBM) [2]. Serotonin may originate from the activity of serotoninergic synapse present in the microenvironment of the tumor, but it was also proposed that GBM cells can gain the ability to produce and secrete their own serotonin ([179]; and see also infra, GBM paragraph). MDD is a common comorbidity for GBM and is known to promote disease progression. MDD and GBM share several pathophysiological pathways, including dysfunction of the serotoninergic system. Due to these overlapping molecular pathways, the benefits of antidepressant treatment on GBM progression are unclear and need further study [180].

Metabolic encephalopathies are a group of neurological disorders not related to primary CNS structural damage. The classification distinguishes those due to lack of glucose, oxygen, or metabolic cofactors from those due to peripheral organ dysfunction [181]. Among the seconds, hepatic encephalopathy (HE) is a consequence of late-stage liver disease that can result from multiple causes, including cirrhosis. Several neurological symptoms are associated with HE, including depressive mood [182]. Abnormal serotonin production was reported in patients with HE, suggesting an involvement of this monoamine in mood disorders associated with this encephalopathy [183,184].

Toxic encephalopathies are caused by acute or chronic exposures to various substances and pollutants that can act as neurotoxicants (see also paragraph 2 for the effects of pollutants on the serotoninergic system). They are characterized by several symptoms, including an altered mental status, seizures, and depressive mood. Toxic encephalopathy was described in patients following co-administration of the dye methylene blue to enable pre-operative visualization of parathyroid glands and SSRI. This dye being a potent MAO-A inhibitor, severe serotonin toxicity (or serotonin syndrome) was suggested [185]. Most cases of serotonin toxicity involve an overdose of serotonin-elevating drugs, monoamine-oxidase inhibitors, serotonin-norepinephrine reuptake inhibitors (SNRIs), and SSRIs.

Major depressive disorders (MDD) are psychiatric illnesses with an etiology determined by a complex set of influences (genetic, social, and environmental). Despite advances in the understanding of the etiology and pathophysiology of MDD [186], currently, no established mechanism can explain all facets of the disease. Among the neurophysiological theories of this disease, the monoamine hypothesis proposes a deficiency of central monoamine systems, including the serotoninergic [187]. Many antidepressant drugs act by inhibiting the reuptake of one or more monoamine neurotransmitters or by an increase in neurotransmitters release and thus improve the neurotransmission system altered in MDD. For example, SSRIs, some of the most commonly prescribed drugs worldwide, inhibit serotonin uptake through the blockage of neuronal and astrocytic SERT, and the subsequent enhancement of synaptic serotonin levels is known to act on 5-HT receptors that mediate antidepressant response. Moreover, reduced serotoninergic neurotransmission is a hypothesis to explain the etiology of suicide [188]. Two other hypotheses, the neurotrophic and neurogenic hypotheses, have been proposed to explain the role of serotonin in the pathophysiology of depression. These hypotheses are based on the fact that 5-HT receptors and 5-HT signaling are involved in regulating the levels of both neurotrophic factors (i.e., BDNF, VEGF, FGF2, IGF1) and adult neurogenesis in the subgranular zone of the dentale gyrus in the hippocampus [189]. In a previous review, we reported that many environmental chemical pollutants had been related to the etiology of MDD [17]. Several epidemiological studies suggest that exposure to BPA [190] phthalates [191,192], heavy metals [190], PAH [192], pesticides [190], and airborne pollutants [193] contribute to an increased prevalence of MDD. Moreover, in mice, early life exposure to BPA dose, representative of human exposure levels, induces depressive-like behavior specific to F1 generation adult males, associated with a reduction in whole hippocampal serotonin levels [194]. Interestingly, hippocampal and frontal cortex serotonin levels were reduced in a stress-sensitive rat model of depression following chronic O_3_ exposure [49].

Migraine is an episodic neurobehavioral disorder with complex pathophysiology, which depends upon gene-environmental interactions. Studies have implicated serotonin and its signaling pathways in the pathophysiology of migraines, and the mainstay acute treatment for migraines is a class of drugs, which act on serotonin receptors, called triptans. Although migraine was proposed to be a low serotonin syndrome, it was recently suggested that migrainers have a low brain serotonin level between attacks and that this level elevates as a consequence of migraine attacks [195,196]. Moreover, a recent study of 117 migraine Danish families suggests that dysfunction in the 5-HT2 receptor-mediated signaling pathway is part of migraine pathophysiology [197]. The function of serotonin in migraine pathophysiology is complex, depending on the site of action and the receptor subtype it activates [196]. The influence of environmental factors on the attacks of migraine led to extensive debate over the past decades, and determining the triggering factors is crucial to prevent this disorder. Chemical exposure and specific environmental irritants are well-known triggers of the attacks of migraine [198]. In a study made in Seoul (South Korea), higher air pollutant levels from traffic combustion sources were associated with the risk of migraine, especially on high-temperature days [199]. Air fresheners emit volatile organic compounds (terpenes, benzene, toluene, …) and semi-volatile organic compounds (such as phthalates) that contribute to indoor hazardous air pollutants associated with migraine headaches [200]. Copper toxicity in women that experience migraine three times more frequently than men and tobacco smoke were also associated with migraine attacks [201,202]. A multibehavioral model of migraines in rats developed based on clinical diagnostic criteria from the International Classification of Headache Disorders demonstrates that BPA exposure can exacerbate migraine-like behaviors and alter mRNA levels of a number of nociception-related genes [203].

## 6. Cancer

The most common primary malignant brain tumor in adults is the grade IV glioma, GBM. It has a poor prognosis with an estimated overall survival time of 16–18 months. During the last 30 years, the incidence of GBM in France increased about four times. Better diagnostic imaging and population aging are not sufficient to explain such a rise. Among exposures involved in brain tumors (ionizing radiations) [204], carbamate pesticides have been recently implicated in excess of CNS tumors in farmers [205]. A high number of GBM cases was also observed in areas probably contaminated by aluminum [206]. Molecular mechanisms linking chemical exposure to GBM are unknown [207]. However, it is possible that such exposure may interact with the serotonin network. Indeed, serotonin activates adult neurogenesis and gliogenesis [179,208,209,210] by acting on neural stem cells whose characteristics are similar to initiating cells from which GBM derives [211,212]. Several human GBM cell lines express 5-HT receptors such as 5-HT7 [213,214]. Other 5-HT receptors have been found to be specifically (i.e., 5-HT1E, 5-HT1Dα) expressed in human glioma cell lines or at higher rates (i.e., 5-HT2) than in normal astrocytes [215]. These data suggest that serotonin activates cell proliferation, migration, and invasion of glioma cells without acting on a particular receptor [215]. Knowing that serotonin receptors interact with MAPK and Akt cascades reinforces their involvement in GBM growth [7,8]. Rat C6 glioma cells treated with 5-HT2A agonists exhibit increased proliferation and migration [216,217]. This highlights the potential for serotonin receptor activation to promote GBM growth and invasion [179]. This is in line with PET scanning observation showing serotonin binding to various receptors [218] and tryptophan uptake correlated with decreased survival of patients [219]. The tumor source of serotonin may come from the platelet aggregation of the thrombotic environment of GBM. Serotonin would not only act directly on GBM cell growth but also on angiogenesis by enhancing endothelial cell growth [220,221].

To conclude, our lack of knowledge about etiological factors prevents us from establishing whether they affect glioma genesis by impacting directly on the serotoninergic system. However, as presented above, the serotonin receptors seem to be involved in all aspects of glioma growth and reflect by their variety the characteristic high heterogeneity of GBM tumors [222]. Despite their variety, it is possible then to consider the serotoninergic system, through its receptors, as a therapeutical target for limiting glioma growth by using specific inhibitors [223].

## 7. Discussion and Future Directions

The incidence of brain pathologies has increased in recent years [224], and a link with environmental pollutants is suspected [17]. Environmental factors through epigenetic markings could exacerbate the genetic susceptibility of some patients. Interestingly, a decline in a child’s intelligence quotient is also observed [225], and chemical pollution could be a factor contributing to this decline. The developing nervous system of a fetus and newborn is susceptible to the deleterious effects of environmental neurotoxic substances, and perinatal exposure could result in the later development of diseases, a hypothesis known as the developmental origin of health and diseases [16]. As discussed in this review, some of these environmental pollutants affect the serotoninergic system and could be the source of a silent pandemic of neurodevelopmental toxicity.

The comorbidity frequently present between some neuropathies can be understood by the fact that these diseases share, at least in part, a common etiology involving the serotoninergic system. Observations suggest that abnormalities of the serotoninergic system during prenatal and early postnatal development of the CNS may result in a predisposition of these children to brain disorders [19]. A link between brain diseases and the inflammatory processes was proposed to interact in a complex way with serotoninergic pathways [226]. We have recently proposed that a common part between cerebral neuropathies could be inflammatory processes in which connexin 43 (Cx43)- and pannexin-based channels seem to be involved [227]. On the other hand, through the action on some of its receptor subtypes, serotonin could regulate Cx expression and/or function, leading to a complex interplay between these cellular mechanisms [228].

Studies have indicated that environmental chemicals (bisphenols, phthalates, persistent organic pollutants, heavy metals, and pesticides) exposition during various stages of life could significantly affect the human gut microbiome and the host health. One open question is to what extent, and how, gut microbiome mediates the brain disorders-causing effects of the pollutants [229]. Interestingly, the brain-gut axis is a bidirectional communication network in which serotonin acts at both ends. Moreover, it was suggested that the gut microbiome impacts the host serotoninergic system through its tryptophan metabolism [230].

As suggested by studies carried out on the sudden infant death syndrome (SIDS), the number of pathologies whose etiology involves a defect in the serotoninergic system would be greater, and environmental pollutants would be partly responsible for the increase in their prevalence [231,232]. The number of CNS pathologies, in which part of the etiology is a disruption of the serotoninergic system under the influence of environmental pollutants, is probably underestimated. Most of these pollutants that accumulate in the environment due to agricultural, industrial, and urban activities have a long persistence, can still be widely found in soils, in water, in the air, and in various animal species, decades after their use has been discontinued. Human exposure to these pollutants can occur by several routes, including ingestion of contaminated foods, skin absorption, or through respiration and accidental contamination. However, despite overwhelming evidence of the impact of environmental pollutants on human health, restrictive political decisions to limit or eliminate this pollution have still not been taken. Consequently, in the short term, the availability of a large array of pharmacological tools acting on the serotoninergic system makes it possible to use some of them in the treatment of chemical pollutants-related brain diseases.

What is really known about environmental risks? Epidemiological and clinical studies suggest a correlation between human exposition to environmental pollutants and the incidence of brain pathologies. However, as correlation does not necessarily mean causation, these observational findings need to be completed with experimental studies on the biological mechanisms. In the future, new in vitro models using pluripotent stem cells, brain organoids, and culture of neuronal cells derived from patients may help to ascertain in these pathologies the disruption of the serotoninergic system under the influence of environmental pollutants.
